# Long-Term Health Effects of Curative Therapies on Heart, Lungs, and Kidneys for Individuals with Sickle Cell Disease Compared to Those with Hematologic Malignancies

**DOI:** 10.3390/jcm11113118

**Published:** 2022-05-31

**Authors:** Courtney D. Fitzhugh, Emmanuel J. Volanakis, Ombeni Idassi, Josh A. Duberman, Michael R. DeBaun, Debra L. Friedman

**Affiliations:** 1 Cellular and Molecular Therapeutics Branch, National Heart, Lung, and Blood Institute, NIH, Building 10, MSC 1589, Bethesda, MD 20814, USA; 2 Department of Pediatrics, Vanderbilt-Meharry Center of Excellence in Sickle Cell Disease, Vanderbilt University Medical Center, 2525 West End Ave., Suite 750 VIGH, Nashville, TN 37203, USA; emmanuel.volanakis@vumc.org (E.J.V.); oidassi19@email.mmc.edu (O.I.); m.debaun@vumc.org (M.R.D.); 3 Office of Research Services, Office of the Director, NIH Library, NIH, Building 10, MSC 1150, Bethesda, MD 20892, USA; dubermaj@ors.od.nih.gov; 4 Department of Pediatrics, Vanderbilt-Ingram Cancer Center, Vanderbilt University Medical Center, 395 Preston Research Building, 2220 Pierce Ave., Nashville, TN 37232, USA; debra.l.friedman@vumc.org

**Keywords:** sickle cell disease, hematologic malignancies, hematopoietic stem cell transplant, heart, lung, kidney

## Abstract

The goal of curing children and adults with sickle cell disease (SCD) is to maximize benefits and minimize intermediate and long-term adverse outcomes so that individuals can live an average life span with a high quality of life. While greater than 2000 individuals with SCD have been treated with curative therapy, systematic studies have not been performed to evaluate the long-term health effects of hematopoietic stem cell transplant (HSCT) in this population. Individuals with SCD suffer progressive heart, lung, and kidney disease prior to curative therapy. In adults, these sequalae are associated with earlier death. In comparison, individuals who undergo HSCT for cancer are heavily pretreated with chemotherapy, resulting in potential acute and chronic heart, lung, and kidney disease. The long-term health effects on the heart, lung, and kidney for children and adults undergoing HSCT for cancer have been extensively investigated. These studies provide the best available data to extrapolate the possible late health effects after curative therapy for SCD. Future research is needed to evaluate whether HSCT abates, stabilizes, or exacerbates heart, lung, kidney, and other diseases in children and adults with SCD receiving myeloablative and non-myeloablative conditioning regimens for curative therapy.

## 1. Introduction

Sickle cell disease (SCD) is an inherited disease caused by a point mutation and is associated with early mortality. Within the United States, an estimated 1 of every 365 babies of African descent is born with SCD each year, with 104,000 to 138,900 people affected [1]. Children and adults with SCD experience severe and progressive organ disease, stroke, pulmonary hypertension, cardiomyopathy, and kidney failure [2]. The medical costs of SCD are massive, with total lifetime costs exceeding USD 460,000 for patients who live to be 45 years of age [3].

While mortality for children with SCD has improved substantially over the past 4 decades, with >99% of those born in high-resource settings now surviving to 18 years of age [4,5,6], adults continue to die prematurely. Over the last three decades, the life expectancy of adults with SCD has not significantly changed. In a pooled analysis of 300 individuals with SCD after adjustment for entry age into the cohort, the median survival ages were 48.0 years for individuals with phenotypes HbSS/HbSβ0 thal/HbSD and 54.7 years for HbSC/HbSβ+ thal [7]. In addition to a shortened lifespan, individuals with SCD have a significant decrease in quality of life compared to individuals without SCD [8]. The decrease in quality of life is heavily influenced by progressive heart, lung, and kidney disease. We and others have shown that adults with SCD and comorbid organ dysfunction are more likely to die at least 20 years earlier than the general population [9,10,11,12,13,14,15,16,17,18,19]. Proof of principle that hematopoietic stem cell transplant (HSCT) could cure SCD occurred in 1984 when a patient with co-existing acute leukemia was cured of both [20]. Since then, myeloablative HLA-matched sibling HSCT has emerged as the standard transplant option for eligible children [21]. HLA-matched sibling HSCT is also highly efficacious in adults using a non-myeloablative approach [22,23,24]. As most patients do not have an HLA-matched sibling donor, alternative curative therapy options, including haploidentical HSCT with post-transplant cyclophosphamide, gene therapy, and gene editing, are increasingly available with impressive results [25,26,27,28,29,30]. Given the range of variably intense curative treatment options available for children and adults with SCD, maximizing benefit while minimizing the short, intermediate, and long-term health risks requires data-driven personalized health care. Individuals with SCD who undergo curative therapy with HSCT, gene editing, or gene therapy differ from individuals who undergo HSCT for malignancies. Except for hydroxyurea, individuals with SCD do not receive myeloablative chemotherapy, radiation therapy, or immunotherapy before HSCT. Further, individuals with SCD have a lifetime predisposition to developing subclinical or overt organ damage, including the heart, lung, and kidney, due to their underlying disease. In the general population, consensus guidelines, based primarily on expert opinion and large observational studies for long-term follow-up after HSCT therapy, were developed without considering SCD [31,32,33,34]. Recommendations included post-curative screening for sequelae in heart, lungs, kidneys, brain, liver, eyes, endocrine organs, teeth, skin, and mucous membranes, as well as for secondary malignancies (Table 1). These recommendations arise from the Children’s Oncology Group [34], National Marrow Donor Program (BeTheMatchClinical.org/guidelines, accessed on 1 July 2021), and expert opinion [35]. To our knowledge, few studies have evaluated the adherence to these guidelines, and it is unclear whether recommendations established following HSCT for malignancy are fully extrapolatable after curative therapy for SCD.

To address this gap in knowledge, in 2016, the Second Pediatric Blood and Marrow Transplant International Consensus Conference on Late Effects after HCT issued a consensus-based statement on health outcomes for children with SCD and thalassemia treated with HSCT [37]. Based on their literature review and research priorities, experts developed consensus-based guidelines in 2018 for monitoring organ systems, graft-versus-host disease (GVHD), health-related quality of life, nutritional and metabolic function, health care utilization, and malignancy screening [36]. Table 1 summarizes the long-term follow-up guidelines from the pediatric consortium described above for SCD [36] and for contrast, those established for post-HSCT surveillance among patients with malignancy. While greater than 2000 individuals with SCD have been treated with curative therapy to date, systematic studies have not been performed to evaluate the long-term health effects of HSCT. Such studies will be requisite to inform evidence-based long-term follow-up guidelines. In this paper, we first review the studies that describe the impact of HSCT on the heart, lung, and kidney, mostly in cancer survivors, followed by review of the much more limited data available primarily for children but also adults who have received curative therapy for SCD.

## 2. Materials and Methods

Various literature search methods were used to identify relevant studies involving the long-term health outcomes for patients with cancer and SCD during the summer of 2020, and an updated manual search was performed in July 2021. Retrieved answer sets from PubMed^®^ (National Library of Medicine) and Embase^®^ (Elsevier) searches were analyzed, and relevant articles were evaluated, as well as citations in those articles. 

The term “late effects” was not a controlled vocabulary term in Embase^®^ or PubMed at the time of this study. “Late effects” was searched as a text phrase, and the concept “late effects” was also attributed to relevant articles after analysis.

Embase search strategies were predominantly based on therapy terms using the Emtree^®^ controlled vocabulary, as shown in these two examples:

(1) ((“allogeneic stem cell transplantation”/exp/mj OR “allogeneic hematopoietic stem cell transplantation”/exp/mj OR “allogeneic peripheral blood stem cell transplantation”/exp/mj OR “autologous stem cell transplantation”/exp/mj OR “cord blood stem cell transplantation”/exp/mj OR “hematopoietic stem cell transplantation”/exp/mj OR “nonmyeloablative stem cell transplantation”/exp/mj OR “peripheral blood stem cell transplantation”/exp/mj) AND “late effects”) AND (“article”/it OR “article in press”/it OR “review”/it)
  and
(2) ((“allogeneic stem cell transplantation”/exp/mj OR “allogeneic hematopoietic stem cell transplantation”/exp/mj OR “allogeneic peripheral blood stem cell transplantation”/exp/mj OR “autologous stem cell transplantation”/exp/mj OR “cord blood stem cell transplantation”/exp/mj OR “hematopoietic stem cell transplantation”/exp/mj OR “nonmyeloablative stem cell transplantation”/exp/mj OR “peripheral blood stem cell transplantation”/exp/mj) AND (“sickle cell anemia”/exp/mj NOT (“sickle cell crisis”/exp OR “sickle cell trait”/exp))) AND “late effects”

In PubMed, several keyword-based search strategies were used. Therapy terms “myeloablative” or “non-myeloablative” were searched along with keywords for organ systems, with and without sickle cell terms. Two other examples of PubMed search strategies included were:

(1) ((sickle cell disease) OR (sickle cell anemia)) AND ((stem cell transplant) OR (bone marrow transplant) OR (hematopoietic cell transplant)) AND (late effects)
  and
(2) ((sickle cell disease) OR (sickle cell anemia)) AND ((stem cell transplant) OR (bone marrow transplant) OR (hematopoietic cell transplant)) AND (kidney OR renal).

## 3. Results

### 3.1. Adverse Long-Term Health Outcomes in Cancer Survivors Treated with HSCT

In the 1980s, curative therapies rapidly evolved in the pediatric oncology field. While there was an encouraging increase in survival, systematic follow-up of the individuals revealed adverse long-term health challenges. Therefore, a new field emerged: survivorship in pediatric oncology. Most deaths in cancer patients treated with HSCT happen within the first 2 years. While long-term survival for patients who survive 2 years is approximately 80–90%, life expectancy remains lower than in the general population [38,39,40,41]. A study involving 1022 survivors transplanted between 1974 and 1998 reported that 66% had at least one chronic condition and 18% had severe or life-threatening conditions, whereas rates were 39% and 8%, respectively, in siblings [42,43]. Long-term follow-up studies have revealed adverse outcomes affecting the heart, lungs, and kidneys. Further, a recent study that assessed mortality in more than 4000 patients over 40 years revealed that leading causes of nonrecurrence-related mortality included pulmonary and cardiovascular disease [41].

#### 3.1.1. Cardiovascular Complications after HSCT in Individuals with Cancer

Cardiovascular (CV) diseases lead to significant morbidity and mortality in HSCT recipients [34,44,45]. HSCT survivors have a 2- to 4-fold increased risk of CV death compared to the general population [43,46,47,48]. Exposure to anthracycline-based chemotherapy and or chest irradiation pre-HSCT, comorbidities (including diabetes mellitus, hypertension, abnormal body composition, and dyslipidemia), pre-HSCT smoking, iron overload, and chronic GVHD are risk factors for the development of late cardiovascular disease [49,50,51,52,53,54]. Long-term HSCT survivors are also at increased risk for multiple cardiovascular risk factors such as hypertension, obesity, dyslipidemia, constrictive pericarditis, congestive heart failure, cardiomyopathy, conduction abnormalities, and valvular heart disease; prior chest radiotherapy increases the risk for many of these complications [43,55]. Recently, Yeh and colleagues reported cardiac comorbidities, older age, hypertension, diabetes, and arrhythmia were associated with increased risk for cardiac toxicity following HSCT; on the other hand, post-transplant cyclophosphamide was not [56].

The incidence of hypertension ranges from 21.4% to 74% in long-term survivors of HSCT [47,57,58]. A study including 1089 HSCT survivors and a mean follow-up of 8.6 years reported that after adjustment for age, race, sex, and body mass index, patients who had received an allogeneic HSCT had a 2-fold risk of hypertension compared to sibling donors or autologous HSCT survivors [59]. Hypertension is related to specific therapies for GVHD (e.g., steroids, calcineurin inhibitors) and to GVHD-induced endothelial damage and proinflammatory cytokine response [60]. Long-term survivors of allogeneic HSCT are more likely to take cardiovascular medications than those who received chemotherapy only (10% versus 1%, *p* < 0.05) [61].

HSCT can also impact heart function. One study noted that cumulative incidence of shortening fraction abnormalities increased from 12% before to 26% at 5 years post-HSCT [62]. With further follow-up post-HSCT, congestive heart failure cumulative incidence rises further. In a study of 1244 patients, the incidence of heart failure at 5 years post-transplant was 4.8%, and increased to 9.1% at 15 years post-HSCT [63,64]. The patients had a 4.5-fold increased risk of congestive heart failure compared to the general population, and the presence of hypertension or diabetes in these survivors led to a 35-fold or 27-fold risk of congestive heart failure, respectively. Female sex and polymorphisms in certain genes that impact anthracycline metabolism have also been associated with post-HSCT congestive heart failure [65]. Mortality is high, as >50% of patients die within 5 years after diagnosis of congestive heart failure [49,63]. Risk factors for late congestive heart failure include anthracycline dose ≥250 mg/m^2^, number of pre-HSCT chemotherapy cycles, and chronic comorbidities [49,66].

Late health effects of HSCT may not be associated with future cardiac disease. Armenian et al. reported that after adjusting for cardiotoxic exposures, the risk for cardiovascular complications in individuals transplanted for malignancy was the same as that seen in conventionally treated cancer survivors [53]. Therefore, the risk for late cardiovascular complications post-HSCT in individuals with cancer may be primarily due to pre-HSCT treatment exposures instead of conditioning-related exposures or other HSCT complications. In agreement with this hypothesis, children and adults without cancer who underwent allogeneic HSCT for transfusion-dependent beta thalassemia did not develop cardiac function abnormalities with a median follow-up of 7 years [67].

Pre-HSCT and conditioning-related radiotherapy together with cardiovascular risk factors (including diabetes, hypertension, and dyslipidemia) may lead to atherosclerosis [68,69,70]. With a median age of 39 years and a median follow-up of 9 years, 6.8% of 145 patients had developed an arterial event, including atherosclerosis, after allogeneic HSCT, and 2.1% after autologous HSCT [71,72]. The cumulative incidence of an arterial event by 25 years post-allogeneic HSCT was 22.1%. The incidence of myocardial ischemia post-HSCT ranges from 1% to 6% [47].

Not surprisingly, the majority of the studies reporting the incidence of CV complications post-HSCT have been in the myeloablative setting. On the other hand, Kersting et al. evaluated 14 patients who underwent a non-myeloablative approach, and the incidence of hypertension was similar at baseline compared to a median of almost 3 years post-HSCT [73]. Therefore, while the non-myeloablative study is small, the incidence of hypertension post-HSCT may be increased in the setting of myeloablative as opposed to non-myeloablative conditioning. The incidence of GVHD with associated use of steroids and renal failure with myeloablative regimens may contribute to the hypertension post-HSCT [74].

Pulmonary hypertension has rarely been reported in pediatric HSCT recipients [75,76,77]; still, the incidence is higher than idiopathic pulmonary arterial hypertension in the general population. Radiation, chemotherapy, and GVHD may contribute to the development of pulmonary hypertension. Because of the subtle symptoms in the early course of the disease, pulmonary hypertension may be underrecognized [78]. Further, as symptoms may be missed early on, pulmonary hypertension presents as a severe disease. Keen alertness of patients at risk and a heightened sense of early symptoms may allow for a timely diagnosis before developing irreversible complications.

#### 3.1.2. Pulmonary Complications after HSCT in Individuals with Cancer

Chronic pulmonary dysfunction post-HSCT can manifest as restrictive or obstructive lung disease, diffusion abnormality, or combination [34,79,80,81,82]. Respiratory complications occur in 25–50% of patients undergoing allogeneic HSCT, contributing to about 50% of HSCT-related deaths [82]. In almost 50% of patients with pulmonary disease, no infectious source is identified. At 5 years or more post-HSCT, the risk of respiratory complications was 1.5-fold higher in HSCT survivors than cancer survivors who did not undergo HSCT [83]. A recent study showed that the incidence of pulmonary toxicity did not change with busulfan versus total body irradiation (TBI)-based conditioning [84].

Non-infectious pulmonary complications include pulmonary hypertension, cryptogenic organizing pneumonia, and bronchiolitis obliterans [40,85,86,87]. Bronchiolitis obliterans is a manifestation of chronic GVHD and is characterized by new-onset fixed airflow obstruction after allogeneic HSCT. The incidence of bronchiolitis obliterans ranges from 2% to 14% post-allogeneic HSCT and has a mortality rate of 50% [88,89,90,91,92,93]. Non-Caucasian race, lower baseline forced expiratory volume in 1 s/forced vital capacity (FEV1/FVC), and presence of chronic GVHD were risk factors for bronchiolitis obliterans.

Unlike bronchiolitis obliterans, cryptogenic organizing pneumonia is a restrictive lung disease due to the interstitial deposition of fibroblasts within alveoli, alveolar ducts, and bronchioles [82]. Cryptogenic organizing pneumonia post-allogeneic HSCT incidence ranges from 1.7% to 10.3% [94,95,96,97]. Patients with cryptogenic organizing pneumonia were more likely to have acute skin GVHD and chronic GVHD involving the oral cavity and gut [94]. The mortality rate associated with cryptogenic organizing pneumonia is 21% [98].

Prior exposure to drugs and radiation including methotrexate, bleomycin, carmustine, cyclophosphamide, busulfan, TBI and mantle radiation, chronic GVHD, and older age at diagnosis are risk factors for the occurrence of late pulmonary fibrosis post-HSCT [40,51,99,100]. The severity and incidence of radiation-induced lung damage are related to the total volume of lung irradiated, the total dose and type of radiation, and the fractionation of that dose [80,101,102]. Myeloablative conditioning has been associated with worsening pulmonary function post-HSCT [103,104]. Abnormal baseline pulmonary function test (PFT) values, older age at the time of HSCT, the occurrence of a respiratory event within 1 year post-HSCT, the timing of HSCT after first complete remission, peripheral blood stem cells, tobacco use, GVHD, gender, cumulative doxorubicin dose, second HSCT, and high-risk hematologic malignancies have been associated with abnormal pulmonary function test values post-allogeneic HSCT [62,105,106,107,108,109,110,111,112]. Further, three studies revealed a more significant detriment in lung function for patients receiving myeloablative than non-myeloablative regimens [113,114,115]. In patients with systemic sclerosis who underwent autologous HSCT with non-myeloablative conditioning, FVC % improved up to 3 years post-HSCT [116]. DLCO % has also been reported to stabilize or improve in other patients undergoing autologous HSCT for autoimmune diseases using non-myeloablative conditioning [117]. These studies confirm that respiratory complications are frequent post-HSCT and suggest that they may be more severe in patients who receive a myeloablative than a non-myeloablative regimen.

#### 3.1.3. Kidney Complications after HSCT in Individuals with Cancer

Estimates of the incidence of acute kidney injury associated with HSCT vary widely, ranging from 10% to 73% of patients [118]. One study including 272 patients who underwent myeloablative HSCT (89% allogeneic, 11% autologous) revealed that 53% of patients developed acute kidney injury, approximately half of whom required dialysis [119]. Myeloablative allogeneic HSCT was associated with a higher incidence of severe kidney failure and need for dialysis than the non-myeloablative group, even when controlling for baseline characteristics such as age and comorbidities [120].

Chronic kidney disease may occur following an acute kidney injury, commonly related to viral nephropathy (BK virus) [121] or calcineurin-induced thrombotic microangiopathy [40]. The incidence of thrombotic microangiopathy post-allogeneic HSCT ranges from 0.5% to 76%, depending on the specific definition [122]. Chronic kidney disease is reported in approximately 4–60% of patients post-HSCT, with risk differing by stage of kidney disease at baseline and type of HSCT [87,123,124,125].

Risk factors for chronic kidney disease post-HSCT include poor pre-HSCT kidney function, pre-transplant chemotherapeutic exposures with ifosfamide and cisplatin, older age, female gender, use of nephrotoxic medications including calcineurin inhibitors, cytomegalovirus treatment and antibiotics, fludarabine administration, a primary diagnosis of multiple myeloma, hypertension, exposure to high-dose radiation, and acute and chronic GVHD [34,51,72,90,123,126,127,128,129,130,131,132,133,134,135,136]. Studies are conflicting regarding whether chronic kidney disease is not [120,137] or is [138,139] associated with decreased survival. Chronic kidney disease occurred in 22% of patients who underwent non-myeloablative conditioning at 4 years post-HSCT [73]. Therefore, chronic kidney disease, including nephrotic syndrome and acute kidney injury, occurs commonly post-HSCT in childhood cancer survivors and may improve with non-myeloablative conditioning.

Nephrotic syndrome occurs in 0.4–8% of patients post-allogeneic HSCT [40,129,140]. In a study of 279 patients transplanted, the incidence of nephrotic syndrome was higher in recipients of peripheral blood stem cell transplant (24%) as compared to those who received a bone marrow graft (3%), possibly due to the increased incidence of chronic GVHD in patients who receive peripheral blood stem cells versus bone marrow [141]. Nephrotic syndrome may also occur more frequently after non-myeloablative than myeloablative conditioning [140].

### 3.2. Long-Term Adverse Health Outcomes Following HSCT for SCD

We review the published data on heart, lung, and kidney function in individuals with SCD following curative therapy.

#### 3.2.1. Cardiovascular Outcomes following Curative Therapy in SCD

Few studies have evaluated the impact of curative therapy on the progression or attenuation of cardiovascular disease (CVD) in SCD. An elevated tricuspid regurgitant velocity, pulmonary and systemic hypertension, and systolic and diastolic dysfunction are risk factors for early mortality in adults with SCD [10,142,143,144,145,146,147]. Unfortunately, the limited data available are primarily in the pediatric setting. Among 63 children who received an HSCT with myeloablative conditioning at a single center, with a median follow-up of 1.5 years, 36.4% had systolic blood pressure >90th percentile before HSCT, compared with 18.2% following HSCT. The percentage of patients with diastolic blood pressure >90th percentile before HSCT was 2.3% before HSCT but rose to 13.6% after HSCT. In addition, five patients were newly diagnosed with hypertension after HSCT (Table 2) [148]. Another report of 18 pediatric patients who underwent non-myeloablative matched related donor HSCT demonstrated that six (33.3%) had hypertension reported at some point during follow-up, and three required antihypertensive therapy. There were no reports of severe hypertension, defined as >95th percentile plus 12 mmHg in children, ≥140/90 mmHg, or requiring antihypertensive therapy in adults [149]. Notably, the mean follow-up was limited, with a mean follow-up of 128.6 weeks (Table 2).

Pulmonary and systemic hypertension may occur after curative therapy due to graft-versus-host disease (GVHD) or calcineurin inhibitors and corticosteroids used to prevent or treat GVHD and renal failure. An elevated tricuspid regurgitant velocity (TRV), defined as at least 2.5 m/s, is associated with pulmonary hypertension and an increased risk of death in SCD [157]. Few single studies, and no multicenter studies, have systematically evaluated the clinical history of TRV after curative therapy. In a single-center study, 17 children underwent reduced-intensity conditioning and received HLA-matched related donor HSCT (Table 2) [156]. One patient had TRV 2.8 m/s before HSCT and trivial to mild tricuspid regurgitation 1 and 2 years after. Of the 11 patients who had follow-up transthoracic echocardiograms (ECHO) at 1 year and the five patients who had ECHO at 2 years, none had evidence of pulmonary hypertension. A more recent study reported 30 adults who underwent non-myeloablative conditioning from a matched related donor [22]. Those with a baseline pre-transplant TRV over 2.5 m/s saw an improvement from a mean of 2.8 m/s before to a mean of 2.3 m/s 3 years after HSCT. These patients also demonstrated an improved 6-min walk test. Together, small studies suggest that curative therapies may improve a risk factor for early mortality in adults with SCD. Large, multicenter studies are indicated in children and adults to validate the results.

While hypercholesterolemia and atherosclerosis are rare in individuals with SCD [158], decreased erythropoietic stress and therefore decreased metabolism after successful curative therapy may increase the long-term risk of cardiovascular disease. There are no studies to date that perform serial assessments of cholesterol levels in patients with SCD who underwent HSCT.

Limited evidence suggests that cardiac function may, in some cases, be adversely affected by HSCT. The multicenter Sickle Transplant Alliance for Research (STAR) registry collected data from 174 patients who received an HSCT between 1993 and 2016 (Table 2) [150]: 75% (131) received cells from a matched related donor within the registry, and 58.1% received myeloablative conditioning. With a median follow-up of 3.2 years, 4.3% of patients with ejection fractions measured pre- and post-HSCT (5 of 116) had a change in ejection fraction from >55% before transplant to ≤55% after. Further, 2.9% of patients with shortening fractions measured pre- and post-HSCT (4 of 136) had a change in shortening fraction from >28% before transplant to ≤28% after. In another study, with a median follow-up of 4.2 years, among 355 participants with SCD, and a median age at HSCT of 10 years, enrolled in the Center for International Blood and Marrow Transplant Research (CIBMTR) registry between 1996 and 2015, the prevalence of congestive heart failure developing after HSCT was about 1% [151]. In a more recent study of 19 patients who underwent myeloablative haploidentical HSCT, fractional shortening was stable at 2 years post-HSCT compared to pre-HSCT [153]. In another small study of 13 children who received myeloablative HLA-matched sibling HSCT and 3 children who underwent reduced-intensity haploidentical HSCT, with a median follow-up of 8.6 years, all patients had normal cardiac function before HSCT; however, the median shortening fraction decreased from 41% (range 34–51%) to 37.5% (28–44%, *p* = 0.001) [152]. However, there was no change in shortening fraction post-HSCT when only evaluating the three patients who received reduced-intensity conditioning.

Sachdev et al. recently reported the ECHO results for individuals with SCD who received non-myeloablative conditioning followed by HLA-matched sibling or haploidentical HSCT [154]. Echocardiograms were analyzed at baseline and 3, 6, and 12 months post-HSCT. After successful HSCT among 44 patients, there were significant improvements in cardiac size, function, and diastolic filling parameters at 3 months, followed by continued, more minor improvements up to 1 year post-HSCT. Sachdev and colleagues also found a mild decrease in the myocardial strain at 3 months through 1 year post-HSCT, possibly from total body irradiation (TBI) or cyclophosphamide (Table 2) [154]. In another study, Saraf et al. reported that left atrial diameter decreased significantly at 1 year post-non-myeloablative HLA-matched sibling HSCT [23]. A pediatric study including 11 pediatric patients with SCD who received myeloablative HSCT reported significantly decreased myocardial strain at a mean of 109 days post-HSCT [155]. However, at 1 year post-HSCT, myocardial strain improved and was comparable to baseline.

#### 3.2.2. Pulmonary Outcomes following Curative Therapy in SCD

Upon evaluating data obtained from the Cooperative Study of SCD, Kassim and colleagues found that in 430 adults who did not receive curative therapy, lower predicted percent forced expiratory volume in 1 s (FEV1%) was associated with an increased hazard ratio for death [159]. Sparse data that follow the trajectory of lung function after curative therapy are available. In a prospective multicenter study conducted between 1991–2000 (Multicenter Investigation of Bone Marrow Transplantation for Sickle Cell Disease), 59 children ≤16 years old underwent HSCT from a matched related donor with myeloablative conditioning (Table 3) [160]. Among the 23 patients who had pulmonary function tests before and after HSCT, there was no difference between pre- and post-HSCT FEV1%. Four of eleven patients who had restrictive lung physiology at baseline went on to have a normal function after HSCT. Eight of the 10 patients who had normal baseline pulmonary function tests continued to have a normal pulmonary function after HSCT; two developed a restrictive pattern. Further, one of the two patients with an obstructive pattern had a normal pulmonary function after HSCT, and the other developed worsened obstructive disease after HSCT. Lastly, there was no significant change in percent predicted diffusing capacity for carbon monoxide (DLCO%) after HSCT.

Similarly, Friedman and colleagues reported 19 children who received myeloablative haploidentical HSCT [153]. At 2 years post-HSCT, lung function was stable to improved. Another study revealed lung function was generally stable or improved after a median of 3 years in five patients who had lung function tests before and after receiving myeloablative or reduced-intensity HSCT [161]. Further, Bhatia et al. reported that in their single-center prospective trial of reduced-toxicity conditioning, FEV1% did not significantly change in 16 children with SCD up to 3 years post-HSCT [152]. Of 13 patients who had lung function tests pre-HSCT, 3 had restrictive lung disease, and 1 had obstructive lung disease [156]. There was no statistically significant change in FEV1% at 1 year (N = 12) and 2 years (N = 11) after HSCT. In addition, at a follow-up of 2–8.5 years after reduced-intensity matched related donor HSCT, Krishnamurti found all 7 patients transplanted had stable lung function post-HSCT [162]. However, data analysis from the CIBMTR demonstrated pulmonary abnormalities developing after HSCT, with a cumulative incidence of about 2% at a median of 4.2 years post-curative therapy (Table 3) [151]. Unrelated donor HSCT was associated with a higher risk of pulmonary abnormalities (HR 5.90, 95% CI 1.14–30.42).

While no studies to date report the effects of myeloablative conditioning on FEV1% for adults with SCD, Saraf et al. reported that FEV1% significantly improved 1 year post-HSCT compared to baseline in 12 patients with SCD who underwent non-myeloablative conditioning (Table 3) [23]. In a more recent study of 122 patients, primarily adults, who received non-myeloablative conditioning with a median follow-up of 4 years [24], FEV1% predicted remained stable throughout follow-up. Further, the proportion of patients with moderate, moderately severe, and severe defects decreased. Therefore, these limited data suggest that FEV1%, a biomarker, when reduced, of early mortality in adults with SCD, may remain stable in children receiving myeloablative and reduced-intensity conditioning and at best improve in adults who undergo non-myeloablative conditioning.

### 3.3. Renal Outcomes following Curative Therapies in SCD

Chronic kidney disease is common among adults with SCD and is associated with early mortality [9,10,11,12,13,18,19], but there is minimal literature on the effect of HSCT on renal function. A report from the CIBMTR described sickle nephropathy developing in 7 (2%) of participants with 4.2 years of follow-up post-HSCT (Table 4) [151]. In a small study with follow-up of 8.2 years, in 13 children with SCD who underwent myeloablative HLA-matched sibling HSCT, mean GFR decreased significantly from 173 ± 56 mL/min/1.73 m^2^ to 101 ± 24 mL/min/1.73 m^2^, *p* = 0.004, and in six children with SCD who received reduced-intensity haploidentical HSCT, mean GFR decreased from 98 ± 33 to 91 ± 47 mL/min/1.73 m^2^, *p* = 0.004 [152]. Matthes-Martin et al. reported the Austrian experience with eight children who received reduced-intensity conditioning for matched related donor HSCT with four years of follow-up [163]. Five patients had sickle nephropathy before HSCT. Four demonstrated normalization of renal volume and structure.

Further, renal function was preserved in seven children post-reduced-intensity matched related donor HSCT [162]. A Canadian report of 18 children who received non-myeloablative conditioning for matched related donor HSCT found a decrease in hyperfiltration two years after HSCT, with a median estimated glomerular filtration rate of 142.3 before transplant and 127.6 mL/min/1.73 m^2^ after [149]. Studies currently do not exist evaluating the incidence of renal failure in adults with SCD who underwent curative therapies.

### 3.4. Summary of Surveillance for Heart, Lung, and Kidney Disease following Curative Therapy for SCD

Despite the research conducted to date in these areas, consensus-based guidelines, particularly for adult patients, have yet to be established. The main risk factors for earlier death in adults with SCD are progressive heart, lung, and kidney disease. Therefore, we have suggested recommendations for heart, lung, and kidney disease surveillance at baseline and following curative therapy (Table 5) based on a review of the late health effects of heart, lung, and kidney disease after HSCT in children and adults with cancer. 

However, given the paucity of data currently available on the late health effects of children and particularly adults who undergo HSCT for SCD and the lack of consensus to date on surveillance, we anticipate that the process of formulating these recommendations will be iterative. With such data, surveillance strategies for progressive organ damage after both myeloablative and non-myeloablative curative therapy in children and adults with SCD will evolve as new data become available, with the long-term goal of evidence- and consensus-based guidelines.

## 4. Discussion

Our understanding of the heart, lung, and kidney disease associated with HSCT is almost exclusively based on cohorts of patients who underwent HSCT for malignancies. As such, they were exposed to pre-transplant chemotherapy, radiation therapy, or both. In addition, HSCT conditioning regimens for cancer differ from those used for SCD. Further, prolonged vaso-occlusion, anemia, inflammation, and hypercoagulability generally lead to subclinical and overt organ damage over the lifetime, including the heart, lung, and kidney, in adults with SCD. Therefore, risk factors for and long-term health effects evaluated in cancer survivors will not necessarily apply to patients with SCD who receive curative therapy. Data in patients treated with curative therapies for SCD are much more limited than those treated with HSCT for malignancy and illustrate the critical need for prospective follow-up of children and adults with SCD who undergo curative therapy.

Heart, lung, and kidney dysfunction in adults with SCD are directly associated with decreased life expectancy. Fortunately for children with SCD, mortality rates have decreased to less than 2% of children before 18 years of age [4,5,6]. Thus, heart, lung, and kidney diseases are not associated with premature death in children. We and others have shown that adults with SCD and heart, lung, and kidney disease are more likely to die at least 20 years earlier than the general population [9,10,11,12,13,14,15,16,17,18,19]. Ultimately, the shortened lifespan of individuals with SCD, attributable to declining heart, lung, and kidney function, must be measured against favorable and unfavorable health outcomes associated with curative therapy. While patient-reported outcomes are essential to collect, studies examining the physiologic impact of curative therapy, including critical parameters such as blood pressure, ECHO, PFTs, and laboratory assessment of kidney disease, are critical to identifying heart and lung and kidney disease at an earlier, asymptomatic state.

New disease-modifying therapies have evolved along with the advances in curative therapy in the last several years. Three new drugs have been FDA-approved to ameliorate acute symptoms associated with SCD [164,165,166]. Defining health outcomes following curative therapy is essential to improve personalized decision making when considering curative versus disease-modifying therapeutic options.

To date, the referenced studies for SCD are small, have a short duration of follow-up, or both. Studies are indicated to evaluate whether HSCT abates, stabilizes, or exacerbates organ dysfunction in patients with SCD, to determine the optimal follow-up time, and to identify risk factors so that a personalized approach to curative therapy for SCD can be pursued. It is imperative to know if HSCT can prolong survival compared to those who receive disease-modifying therapies. In addition, despite an increased number of adults receiving myeloablative chemotherapy in preparation for gene therapy or gene editing, both short- and long-term follow-up is required to determine if these therapies offer a superior risk–benefit ratio to disease-modifying therapy or HSCT.

In conclusion, understanding the short, intermediate, and long-term health tradeoffs will facilitate comparing clinical outcomes for different SCD curative therapies. Studies are critically needed to educate patients, families, and providers about the long-term health effects of curative therapies for SCD and to inform and update guidelines for children and adults who receive curative therapies. Therefore, now is the time to systematically evaluate, with appropriate sample size for statistical significance, organ function in individuals who undergo curative therapies for SCD. With the adequacy of such data, we will meet the longer-term goal for and create new consensus guidelines when more data become available to guide surveillance post-HSCT for individuals with SCD.

## Figures and Tables

**Table 1:** Existing long-term follow-up guidelines for organ function monitoring after HSCT.

System	Pediatric Consortium—SCD-Specific [36]	Children’s Oncology Group [34]	National Marrow Donor Program ^a^	Bhatia [35]
Iron	Serum ferritin and transferrin saturation every 3–6 months- commencing 6 months post-HSCT until normal. Consider cardiac and liver MRI monitoring. Treat until normalized but avoid deferasirox and calcineurin inhibitors together.	Ferritin at 12 months.	Ferritin at 12 months.	Ferritin at 12 months.
Cardiac	Annual Echo and lipid profile every 5 years. Annual BP.	Lipids every 2 years. Annual BP. Echo every 1–5 years.	Same for at risk without time period noted.	Lipids every 2 years. Annual BP. Echo every 1–5 years.
Liver	Assess liver function tests every month through 1 year after HSCT; every 3 months in year 2.	LFTs 1 year post-HSCT.	LFTs 6 months, 1 year, annually.	
Pulmonary	Evaluate PFTs at 3, 6, and 12 months and then yearly for 2 years. Annual PFTs for patients with early compromise or cGVHD until immune-suppressive medications have been stopped. Echo with evaluation of tricuspid regurgitant velocity to rule out pulmonary hypertension at 1 year. Measure pulmonary arterial pressure if tricuspid regurgitant velocity >3 m/s to confirm pulmonary hypertension.	1 year post-HSCT.	PFTs 6 months, 12 months, annually.	1 year post-HSCT.
Neuro	Brain MRI/MRA at 1 and 2 years post-HSCT and then every 2 years as clinically indicated in patients with a history of stroke or moyamoya syndrome pre-HSCT. MRI at 1–2 years post-HSCT in patients with PRES or other neurotoxicity during HSCT. Neurocog assessment.	Neurocog assessment.	MRI without interval specified. Neurocog assessment.	Neurocog assessment.
Renal	Monitor until nephrotoxic therapy is discontinued and yearly for 2 years (BUN, creatinine, electrolytes, GFR, or 24-h creatinine clearance). UA for blood/protein at 1 year. Microalbuminuria testing annually for 2 years. Monitor BP annually.	Renal function for 1 year. Annual UA for protein. Annual BP.	BP and renal function tests at 6 months, 1 year, annually.	Renal function at 1 year. Annual UA for protein. Annual BP.
Ophthalmology	Annually for 1–2 years.	Annually.	Annually.	Annually.
Thyroid	TSH, FT4 at 6 months, 1 year, then annually.	TSH and FT4 annually.	TSH, FT4 at 6 months, 1 year, then annually.	TSH and FT4 annually.
Diabetes	FBS and oral GTT at 1 year.	FBS or HbA1C every 2 years.	FBS or HbA1C 1 year.	FBS or HbA1C every 2 years.
Gonadal	Yearly physical examination. Track Tanner progression. Testosterone, LH, and FSH in males ≥11 years of age, yearly for 2 years. Age-appropriate sperm analysis in male patients. LH, FSH, AMH, and estradiol in female patients ≥11 years of age, at 1 and 2 years post-HSCT.	Tanner every 6 months. Testosterone by age 14. Age-appropriate sperm analysis. LH, FSH, estradiol by age 13.	1 year and annually.	Hormones—no time period.
Growth	Height, weight, body mass index at 6 months, then yearly. Hormone levels for short stature (IGF-1, IGF-B3) and bone age if within growth period by age.	Height, weight, body mass index every 6 months.	Same	
Bone health	Vitamin D (25-OH) level and bone mineral density at 1 year.	Bone mineral density at 1 year.	Bone mineral density at 1 year.	Bone mineral density at 1 year.
Cancer screening		Age-appropriate annual breast exam mammography/MRI if TBI/chest RT—8 years after radiation or age 25 years (whichever occurs last). Colonoscopy every 5 years (minimum) beginning 10 years after RT or age 35 years. Ultrasound and fine-needle aspiration (for those with palpable thyroid nodules).	Age-appropriate annual breast exam mammography/MRI if TBI/chest RT—8 years after radiation or age 25 years (whichever occurs last).	Same for breast. Colonoscopy every 5 years (minimum) beginning 10 years after RT or age 35 years. Ultrasound and fine-needle aspiration (for those with palpable thyroid nodules).
Dental		Annual exam.	Exam 6 months, 1 year, annually.	
Skin and MM		Annual exam.	Exam 6 months, 1 year, annually.	Annual exam.

^a^BeTheMatchClinical.org/guidelines, accessed on 1 July 2021. HSCT: hematopoietic stem cell transplantation; SCD: sickle cell disease; MRI: magnetic resonance imaging; Echo: echocardiogram; BP: blood pressure; LFTs: liver function tests; PFTs: pulmonary function tests; cGVHD: chronic graft-versus-host disease; m/s: meters per second; MRA: magnetic resonance angiogram; PRES: posterior reversible encephalopathy syndrome; Neurocog: neurocognitive; BUN: blood urea nitrogen; GFR: glomerular filtration rate; UA: urinalysis; TSH: thyroid-stimulating hormone; FT4: free thyroxine; FBS: fasting blood sugar; GTT: glucose tolerance test; HbA1C: glycated hemoglobin; LH: luteinizing hormone; FSH: follicle-stimulating hormone; AMH anti-Mullerian hormone; IGF-1: insulin-like growth factor-1; IGF-B3: insulin-like growth factor binding protein-3; 25-OH: 25 hydroxy; TBI: total body irradiation; RT: radiation therapy; MM: mucus membranes.

**Table 2:** Late effects studies involving the heart and cardiovascular system in patients with SCD who receive HSCT.

Reference	N	Regimen	Patient Population	Duration of Follow-Up (Years) *	Comments
None	-	myeloablative	adult	-	-
**HTN**					
Stenger [148]	63	myeloablative	pediatric	1.5	Proportion with diastolic BP exceeding 50th or 90th percentile increased post-HSCT 5 diagnosed with HTN ≥ 6 months post-HSCT
Pedersen [149]	18	non-myeloablative	pediatric	0.35	6 with HTN 0 with severe HTN
**Cardiac Iron**					
None	-	-	-	-	-
**Lipid Levels**					
None	-	-	-	-	-
**Cardiac Function**					
Stenger [150]	174	mostly myeloablative	pediatric	3.2	1 with improved cardiac function 5 with worsening EF 4 with worsening SF
Stenger [151]	355	mostly myeloablative	pediatric	4.2	1% incidence of CHF, associated with older age
Dallas [152]	16	myeloablative or reduced-intensity	pediatric	8.6	Significant decrease in SF for all patients combined but not 3 patients who received reduced-intensity conditioning
Friedman [153]	19	myeloablative	pediatric	2	No change in SF
Sachdev [154]	44	non-myeloablative	adult	1	Significant improvements in cardiac size, function, and filling parameters post-HSCT
Saraf [23]	12	non-myeloablative	adult	1	Decreased left atrial diameter post-HSCT
**Myocardial strain**					
Covi [155]	11	myeloablative	pediatric	up to 2 years	Decreased myocardial strain at 3 months, back to baseline at 1 year post-HSCT
Sachdev [154]	44	non-myeloablative	adult	1	Decreased myocardial strain at 3 months and 1 year post-HSCT
**Tricuspid Regurgitant Velocity**					
Stenger [150]	174	mostly myeloablative	pediatric	3.2	Mean TRV normal in 64 patients post-HSCT
Bhatia [156]	17	reduced-intensity	pediatric	3	1 patient with TRV 2.8 at baseline had trivial to mild TR 1 and 2 years post-HSCT No patients with pulmonary HTN
Hsieh [22]	30	non-myeloablative	adult	3	Patients with a TRV >2.5 had a mean decrease from 2.8 to 2.3 m/s at 3 years post-HSCT

* Duration may represent the entire group transplanted and not necessarily the subpopulation reported. Hypertension: BP measurements > 95th percentile for age, height, and sex, receiving anti-hypertensive medications, or both. Myocardial strain: Describes local lengthening, thickening, and shortening of the myocardium as a measure of regional left ventricular function. Abbreviations: HTN: hypertension, BP: blood pressure; HSCT: hematopoietic stem cell transplant; EF: ejection fraction; SF: shortening fraction; CHF: congestive heart failure; TRV: tricuspid regurgitant velocity; TR: tricuspid regurgitation.

**Table 3:** Late effects studies assessing pulmonary function in patients with SCD who undergo HSCT.

Reference	N	Regimen	Patient Population	Duration of Follow-Up (Years) *	Comments
**Pulmonary Function Tests**					
None	-	myeloablative	adult	-	-
Walters [160]	59	myeloablative	pediatric	3.2	No difference in FEV1% or FVC% pre- and post-HSCT
Stenger [150]	174	mostly myeloablative	pediatric	3.2	No change in pulmonary function in 91 patients pre- and post-HSCT
Stenger [151]	355	mostly myeloablative	pediatric	4.2	2% incidence of pulmonary abnormalities, associated with URD HSCT
Mynarek [161]	5	myeloablative or reduced-intensity	pediatric	3	Lung function stable to improved after HSCT
Dallas [152]	16	myeloablative or reduced-intensity	pediatric	8.6	No change in FEV1%, DLCO%, or FEV1/FVC% post-HSCT
Bhatia [156]	13	reduced-intensity	pediatric	up to 2	No difference in FVC% or FEV1% pre- and post-HSCT
Krishnamurti [162]	7	reduced-intensity	pediatric	2–8.5	No change in pulmonary function after HSCT
Saraf [23]	12	non-myeloablative	adult	1	FEV1% and FVC% improved post-HSCT
Alzarhani [24]	122	non-myeloablative	adult	4	FEV1%, FVC%, DLCO% stable; proportion with moderate, moderately severe, and severe defects decreased

* Duration may represent the entire group transplanted and not necessarily the subpopulation reported. Pulmonary function tests: Noninvasive testing to measure lung volume, rates of flow, gas exchange, and capacity. Abbreviations: FEV1%: percent predicted forced expiratory volume in 1 min; FVC%: percent predicted forced vital capacity; HSCT: hematopoietic stem cell transplant; URD: unrelated donor; DLCO%: percent predicted diffusing capacity for carbon monoxide.

**Table 4:** Late effects studies evaluating kidney function and nephropathy in patients with SCD who receive HSCT.

Reference	N	Regimen	Patient Population	Duration of Follow-Up (Years) *	Comments
**Kidney Function and SCN**					
None	-	myeloablative	adult	-	-
Stenger [151]	355	mostly myeloablative	pediatric	4.2	7 patients developed SCN post-HSCT <3% incidence of renal failure requiring dialysis post-HSCT
Dallas [152]	16	myeloablative or reduced-intensity	pediatric	8.6	Myeloablative group: Significant decrease in CrCl from mean 158 to 103.5 Reduced-intensity group: Significant decrease in CrCl from mean 98 to 91 **
Matthes-Martin [163]	8	reduced-intensity	pediatric	4	4 of 5 patients with SCN before HSCT had normal renal volume and structure after HSCT
Krishnamurti [162]	7	reduced-intensity	pediatric	2–8.5	Renal function preserved post-HSCT
Pederson [149]	18	non-myeloablative	pediatric	2	Decrease in hyperfiltration post-HSCT with median GFR 142.3 before and 127.6 after HSCT *
None	-	non-myeloablative	adults	-	-

* Duration may represent the entire group transplanted and not necessarily the subpopulation reported. ** mL/min/1.73 m^2^. Sickle cell nephropathy: Group of renal complications including hematuria, proteinuria, glomerulopathy, and tubular defects that may occur as a result of sickle cell disease. Abbreviations: SCN: sickle cell nephropathy; HSCT: hematopoietic stem cell transplant; CrCl: creatinine clearance; GFR: glomerular filtration rate.

**Table 5:** Long Term Follow-up Considerations for Heart, Lung, and Kidney Disease Following Curative Therapies for Sickle Cell Disease Based on Available Limited Data.

Heart	Lung	Renal
Echocardiogram annually (including TRV), particularly in patients with an elevated TRV as an HSCT indication, cardiac dysfunction or cardiomyopathy, or as clinically indicated. Less frequent studies can be considered after TRV normalization. Cardiac or pulmonary consultation or both is recommended for those with clinically significant cardiomyopathy, cardiac dysfunction, or pulmonary hypertension. Measure blood pressure yearly. Fasting cholesterol profile every 1–2 years. Consider cardiac MRI monitoring, especially in patients with cardiac iron overload before HSCT.	6. Monitor pulmonary function tests (FEV1, FVC, TLC, DLCO) at 3, 6, and 12 months post-HSCT, then yearly. Less frequent studies can be considered for patients who are not symptomatic, are free of GVHD, and have stable pulmonary function tests. 7. Pulmonary consultation is recommended for those with new or worsening abnormal pulmonary function tests.	8. Check renal function (creatinine, BUN, estimated GFR, electrolytes) until calcineurin inhibitor and other nephrotoxic treatment is stopped and annually thereafter. Less frequent studies can be considered for patients with stable electrolytes and renal function. 9. Cystatin-C measurements should be considered to monitor renal function when available. 10. Testing for microalbuminuria yearly. Less frequent testing can be considered for patients with no albuminuria. More frequent testing may be required for patients on sirolimus. Consider not starting treatment unless documentation that albuminuria is persistent upon repeat testing. 11. Measure blood pressure yearly. 12. Renal consultation is recommended for patients with renal dysfunction.

Abbreviations: TRV: tricuspid regurgitant velocity; HSCT: hematopoietic stem cell transplant; MRI: magnetic resonance imaging; FEV1: forced expiratory volume in 1 min; FVC: forced vital capacity; TLC: total lung capacity; DLCO: diffusing capacity for carbon monoxide; GVHD: graft-versus-host disease; BUN: blood urea nitrogen; GFR: glomerular filtration rate.

## Data Availability

Not applicable.

## References

[1] Hassell K.L. (2010). Population Estimates of Sickle Cell Disease in the U.S. Am. J. Prev. Med..

[2] Thein S.L., Howard J. (2018). How I treat the older adult with sickle cell disease. Blood.

[3] Kauf T.L., Coates T.D., Huazhi L., Mody-Patel N., Hartzema A.G. (2009). The cost of health care for children and adults with sickle cell disease. Am. J. Hematol..

[4] Quinn C.T., Rogers Z.R., McCavit T.L., Buchanan G.R. (2010). Improved survival of children and adolescents with sickle cell disease. Blood.

[5] Couque N., Girard D., Ducrocq R., Boizeau P., Haouari Z., Missud F., Holvoet L., Ithier G., Belloy M., Odièvre M.-H. (2016). Improvement of medical care in a cohort of newborns with sickle-cell disease in North Paris: Impact of national guidelines. Br. J. Haematol..

[6] Telfer P., Coen P., Chakravorty S., Wilkey O., Evans J., Newell H., Smalling B., Amos R., Stephens A., Rogers D. (2007). Clinical outcomes in children with sickle cell disease living in England: A neonatal cohort in East London. Haematologica.

[7] DeBaun M.R., Ghafuri D.L., Rodeghier M., Maitra P., Chaturvedi S., Kassim A., Ataga K.I. (2019). Decreased median survival of adults with sickle cell disease after adjusting for left truncation bias: A pooled analysis. Blood.

[8] McClish D.K., Penberthy L.T., Bovbjerg V.E., Roberts J.D., Aisiku I.P., Levenson J.L., Roseff S.D., Smith W.R. (2005). Health related quality of life in sickle cell patients: The PiSCES project. Health Qual. Life Outcomes.

[9] Fitzhugh C.D., Hsieh M.M., Allen D., Coles W.A., Seamon C., Ring M., Zhao X., Minniti C.P., Rodgers G.P., Schechter A.N. (2015). Hydroxyurea-Increased Fetal Hemoglobin Is Associated with Less Organ Damage and Longer Survival in Adults with Sickle Cell Anemia. PLoS ONE.

[10] Fitzhugh C.D., Lauder N., Jonassaint J.C., Telen M.J., Zhao X., Wright E.C., Gilliam F.R., De Castro L.M. (2009). Cardiopulmonary complications leading to premature deaths in adult patients with sickle cell disease. Am. J. Hematol..

[11] Platt O.S., Brambilla D.J., Rosse W.F., Milner P.F., Castro O., Steinberg M.H., Klug P.P. (1994). Mortality in sickle cell disease. Life expectancy and risk factors for early death. N. Engl. J. Med..

[12] Powars D.R., Elliott-Mills D.D., Chan L., Niland J., Hiti A.L., Opas L.M., Johnson C. (1991). Chronic Renal Failure in Sickle Cell Disease: Risk Factors, Clinical Course, and Mortality. Ann. Intern. Med..

[13] Powars D.R., Chan L.S., Hiti A., Ramicone E., Johnson C. (2005). Outcome of sickle cell anemia: A 4-decade observational study of 1056 patients. Medicine.

[14] Graham J.K., Mosunjac M., Hanzlick R.L., Mosunjac M. (2007). Sickle cell lung disease and sudden death: A retrospective/prospective study of 21 autopsy cases and literature review. Am. J. Forensic Med. Pathol..

[15] Perronne V., Roberts-Harewood M., Bachir D., Roudot-Thoraval F., Delord J.M., Thuret I., Schaeffer A., Davies S.C., Galacteros F., Godeau B. (2002). Patterns of mortality in sickle cell disease in adults in France and England. Hematol. J. Off. J. Eur. Haematol. Assoc./EHA.

[16] Darbari D.S., Kple-Faget P., Kwagyan J., Rana S., Gordeuk V.R., Castro O. (2006). Circumstances of death in adult sickle cell disease patients. Am. J. Hematol..

[17] Lanzkron S., Carroll C.P., Haywood C. (2013). Mortality Rates and Age at Death from Sickle Cell Disease: U.S., 1979–2005. Public Health Rep..

[18] Chaturvedi S., Ghafuri D.L., Jordan N., Kassim A., Rodeghier M., DeBaun M.R. (2018). Clustering of end-organ disease and earlier mortality in adults with sickle cell disease: A retrospective-prospective cohort study. Am. J. Hematol..

[19] McClellan A.C., Luthi J.-C., Lynch J.R., Soucie J.M., Kulkarni R., Guasch A., Huff E.D., Gilbertson D., McClellan W.M., DeBaun M.R. (2012). High one year mortality in adults with sickle cell disease and end-stage renal disease. Br. J. Haematol..

[20] Johnson F.L., Look A.T., Gockerman J., Ruggiero M.R., Dalla-Pozza L., Billings F.T. (1984). Bone-Marrow Transplantation in a Patient with Sickle-Cell Anemia. N. Engl. J. Med..

[21] Gluckman E., Cappelli B., Bernaudin F., Labopin M., Volt F., Carreras J., Simões B.P., Ferster A., Dupont S., de la Fuente J. (2017). Sickle cell disease: An international survey of results of HLA-identical sibling hematopoietic stem cell transplantation. Blood.

[22] Hsieh M.M., Fitzhugh C.D., Weitzel R.P., Link M.E., Coles W.A., Zhao X., Rodgers G.P., Powell J.D., Tisdale J.F. (2014). Nonmyeloablative HLA-Matched Sibling Allogeneic Hematopoietic Stem Cell Transplantation for Severe Sickle Cell Phenotype. JAMA.

[23] Saraf S.L., Oh A.L., Patel P.R., Jalundhwala Y., Sweiss K., Koshy M., Campbell-Lee S., Gowhari M., Hassan J., Peace D. (2016). Nonmyeloablative Stem Cell Transplantation with Alemtuzumab/Low-Dose Irradiation to Cure and Improve the Quality of Life of Adults with Sickle Cell Disease. Biol. Blood Marrow Transplant..

[24] Alzahrani M., Damlaj M., Jeffries N., Alahmari B., Singh A., Rondelli D., Tisdale J.F., Saraf S.L., Hsieh M.M. (2021). Non-myeloablative human leukocyte antigen-matched related donor transplantation in sickle cell disease: Outcomes from three independent centres. Br. J. Haematol..

[25] De la Fuente J., Dhedin N., Koyama T., Bernaudin F., Kuentz M., Karnik L., Socié G., Culos K.A., Brodsky R.A., DeBaun M.R. (2018). Haploidentical Bone Marrow Transplantation with Post-Transplantation Cyclophosphamide Plus Thiotepa Improves Donor Engraftment in Patients with Sickle Cell Anemia: Results of an International Learning Collaborative. Biol. Blood Marrow Transplant..

[26] Bolaños-Meade J., Cooke K.R., Gamper C.J., Ali S.A., Ambinder R.F., Borrello I.M., Fuchs E.J., Gladstone D.E., Gocke C.B., Huff C.A. (2019). Effect of increased dose of total body irradiation on graft failure associated with HLA-haploidentical transplantation in patients with severe haemoglobinopathies: A prospective clinical trial. Lancet Haematol..

[27] Saraf S.L., Oh A.L., Patel P.R., Sweiss K., Koshy M., Campbell-Lee S., Gowhari M., Jain S., Peace D., Quigley J.G. (2018). Haploidentical Peripheral Blood Stem Cell Transplantation Demonstrates Stable Engraftment in Adults with Sickle Cell Disease. Biol. Blood Marrow Transplant..

[28] Ribeil J.-A., Hacein-Bey-Abina S., Payen E., Magnani A., Semeraro M., Magrin E., Caccavelli L., Neven B., Bourget P., El Nemer W. (2017). Gene Therapy in a Patient with Sickle Cell Disease. N. Engl. J. Med..

[29] Esrick E.B., Lehmann L.E., Biffi A., Achebe M., Brendel C., Ciuculescu M.F., Daley H., MacKinnon B., Morris E., Federico A. (2021). Post-Transcriptional Genetic Silencing of *BCL11A* to Treat Sickle Cell Disease. N. Engl. J. Med..

[30] Frangoul H., Altshuler D., Cappellini M.D., Chen Y.-S., Domm J., Eustace B.K., Foell J., De La Fuente J., Grupp S., Handgretinger R. (2021). CRISPR-Cas9 Gene Editing for Sickle Cell Disease and β-Thalassemia. N. Engl. J. Med..

[31] Hashmi S.K., Bredeson C., Duarte R.F., Farnia S., Ferrey S., Fitzhugh C., Flowers M.E., Gajewski J., Gastineau D., Greenwald M. (2017). National Institutes of Health Blood and Marrow Transplant Late Effects Initiative: The Healthcare Delivery Working Group Report. Biol. Blood Marrow Transplant..

[32] Hamblin A., Greenfield D.M., Gilleece M., Salooja N., Kenyon M., Morris E., Glover N., Miller P., Braund H., Peniket A. (2017). Provision of long-term monitoring and late effects services following adult allogeneic haematopoietic stem cell transplant: A survey of UK NHS-based programmes. Bone Marrow Transplant..

[33] Majhail N.S., Rizzo J.D., Lee S.J., Aljurf M., Atsuta Y., Bonfim C., Burns L.J., Chaudhri N., Davies S., Okamoto S. (2012). Recommended screening and preventive practices for long-term survivors after hematopoietic cell transplantation. Bone Marrow Transplant..

[34] Chow E.J., Anderson L., Baker K.S., Bhatia S., Guilcher G.M., Huang J.T., Pelletier W., Perkins J.L., Rivard L.S., Schechter T. (2016). Late Effects Surveillance Recommendations among Survivors of Childhood Hematopoietic Cell Transplantation: A Children’s Oncology Group Report. Biol. Blood Marrow Transplant..

[35] Bhatia S., Armenian S.H., Landier W. (2017). How I monitor long-term and late effects after blood or marrow transplantation. Blood.

[36] Shenoy S., Gaziev J., Angelucci E., King A., Bhatia M., Smith A., Bresters D., Haight A.E., Duncan C.N., de la Fuente J. (2018). Late Effects Screening Guidelines after Hematopoietic Cell Transplantation (HCT) for Hemoglobinopathy: Consensus Statement from the Second Pediatric Blood and Marrow Transplant Consortium International Conference on Late Effects after Pediatric HCT. Biol. Blood Marrow Transplant..

[37] Shenoy S., Angelucci E., Arnold S.D., Baker K.S., Bhatia M., Bresters D., Dietz A.C., De La Fuente J., Duncan C., Gaziev J. (2017). Current Results and Future Research Priorities in Late Effects after Hematopoietic Stem Cell Transplantation for Children with Sickle Cell Disease and Thalassemia: A Consensus Statement from the Second Pediatric Blood and Marrow Transplant Consortium International Conference on Late Effects after Pediatric Hematopoietic Stem Cell Transplantation. Biol. Blood Marrow Transplant..

[38] Martin P.J., Counts G.W., Appelbaum F.R., Lee S.J., Sanders J.E., Deeg H.J., Flowers M.E., Syrjala K.L., Hansen J.A., Storb R.F. (2010). Life Expectancy in Patients Surviving More Than 5 Years After Hematopoietic Cell Transplantation. J. Clin. Oncol..

[39] Wingard J.R., Majhail N.S., Brazauskas R., Wang Z., Sobocinski K.A., Jacobsohn D., Sorror M.L., Horowitz M.M., Bolwell B., Rizzo J.D. (2011). Long-Term Survival and Late Deaths After Allogeneic Hematopoietic Cell Transplantation. J. Clin. Oncol..

[40] Giaccone L., Felicetti F., Butera S., Faraci D., Cerrano M., Vici M.D., Brunello L., Fortunati N., Brignardello E., Bruno B. (2020). Optimal Delivery of Follow-Up Care After Allogeneic Hematopoietic Stem-Cell Transplant: Improving Patient Outcomes with a Multidisciplinary Approach. J. Blood Med..

[41] Bhatia S., Dai C., Landier W., Hageman L., Wu J., Schlichting E., Siler A., Funk E., Hicks J., Bosworth A. (2021). Trends in Late Mortality and Life Expectancy After Allogeneic Blood or Marrow Transplantation Over 4 Decades. JAMA Oncol..

[42] Sun C.-L., Francisco L., Kawashima T., Leisenring W., Robison L.L., Baker K.S., Weisdorf D.J., Forman S.J., Bhatia S. (2010). Prevalence and predictors of chronic health conditions after hematopoietic cell transplantation: A report from the Bone Marrow Transplant Survivor Study. Blood.

[43] Armenian S.H., Chow E. (2013). Cardiovascular disease in survivors of hematopoietic cell transplantation. Cancer.

[44] Gavriilaki E., Gkaliagkousi E., Grigoriadis S., Anyfanti P., Douma S., Anagnostopoulos A. (2019). Hypertension in hematologic malignancies and hematopoietic cell transplantation: An emerging issue with the introduction of novel treatments. Blood Rev..

[45] Duléry R., Mohty R., Labopin M., Sestili S., Malard F., Brissot E., Battipaglia G., Médiavilla C., Banet A., Van de Wyngaert Z. (2021). Early Cardiac Toxicity Associated with Post-Transplant Cyclophosphamide in Allogeneic Stem Cell Transplantation. Cardio Oncol..

[46] Chow E.J., Mueller B.A., Baker K.S., Cushing-Haugen K.L., Flowers M.E., Martin P.J., Friedman D.L., Lee S.J. (2011). Cardiovascular Hospitalizations and Mortality Among Recipients of Hematopoietic Stem Cell Transplantation. Ann. Intern. Med..

[47] Scott J., Armenian S., Giralt S., Moslehi J., Wang T., Jones L.W. (2015). Cardiovascular disease following hematopoietic stem cell transplantation: Pathogenesis, detection, and the cardioprotective role of aerobic training. Crit. Rev. Oncol..

[48] Tuzovic M., Mead M., Young P.A., Schiller G., Yang E.H. (2019). Cardiac Complications in the Adult Bone Marrow Transplant Patient. Curr. Oncol. Rep..

[49] Armenian S.H., Sun C.-L., Francisco L., Steinberger J., Kurian S., Wong F.L., Sharp J., Sposto R., Forman S.J., Bhatia S. (2008). Late Congestive Heart Failure After Hematopoietic Cell Transplantation. J. Clin. Oncol..

[50] Armenian S.H., Sun C.-L., Mills G., Teh J.B., Francisco L., Durand J.-B., Wong F.L., Forman S.J., Bhatia S. (2010). Predictors of Late Cardiovascular Complications in Survivors of Hematopoietic Cell Transplantation. Biol. Blood Marrow Transplant..

[51] Lawitschka A., Peters C. (2018). Long-term Effects of Myeloablative Allogeneic Hematopoietic Stem Cell Transplantation in Pediatric Patients with Acute Lymphoblastic Leukemia. Curr. Oncol. Rep..

[52] Rotz S.J., Ryan T.D., Hlavaty J., George S.A., El-Bietar J., Dandoy C. (2017). Cardiotoxicity and cardiomyopathy in children and young adult survivors of hematopoietic stem cell transplant. Pediatr. Blood Cancer.

[53] Armenian S.H., Chemaitilly W., Chen M., Chow E., Duncan C.N., Jones L.W., Pulsipher M.A., Remaley A.T., Rovo A., Salooja N. (2016). National Institutes of Health Hematopoietic Cell Transplantation Late Effects Initiative: The Cardiovascular Disease and Associated Risk Factors Working Group Report. Biol. Blood Marrow Transplant..

[54] Chow E.J., Wong K., Lee S.J., Cushing-Haugen K.L., Flowers M.E., Friedman D.L., Leisenring W.M., Martin P.J., Mueller B.A., Baker K.S. (2014). Late Cardiovascular Complications after Hematopoietic Cell Transplantation. Biol. Blood Marrow Transplant..

[55] Murbraech K., Wethal T., Smeland K.B., Holte H., Loge J.H., Holte E., Rösner A., Dalen H., Kiserud C.E., Aakhus S. (2016). Valvular Dysfunction in Lymphoma Survivors Treated with Autologous Stem Cell Transplantation. JACC Cardiovasc. Imaging.

[56] Yeh J.C., Whited L.K., Saliba R.M., Rondon G., Banchs J., Shpall E.J., Champlin R.E., Popat U.R. (2021). Cardiac Toxicity after Matched Allogeneic Hematopoietic Cell Transplantation in the Post-Transplant Cyclophosphamide Era. Blood Adv..

[57] Abou-Mourad Y.R., Lau B.C., Barnett M.J., Forrest D.L., Hogge D., Nantel S.H., Nevill T.J., Shepherd J.D., Smith C.A., Song K.W. (2009). Long-term outcome after allo-SCT: Close follow-up on a large cohort treated with myeloablative regimens. Bone Marrow Transplant..

[58] Majhail N.S., Challa T.R., Mulrooney D.A., Baker K.S., Burns L.J. (2009). Hypertension and Diabetes Mellitus in Adult and Pediatric Survivors of Allogeneic Hematopoietic Cell Transplantation. Biol. Blood Marrow Transplant..

[59] Baker K.S., Ness K.K., Steinberger J., Carter A., Francisco L., Burns L.J., Sklar C., Forman S., Weisdorf D., Gurney J.G. (2006). Diabetes, hypertension, and cardiovascular events in survivors of hematopoietic cell transplantation: A report from the bone marrow transplantation survivor study. Blood.

[60] DeFilipp Z., Duarte R.F., Snowden J.A., Majhail N.S., Greenfield D.M., Miranda J.L., Arat M., Baker K.S., Burns L.J., Duncan C.N. (2016). Metabolic Syndrome and Cardiovascular Disease after Hematopoietic Cell Transplantation: Screening and Preventive Practice Recommendations from the CIBMTR and EBMT. Biol. Blood Marrow Transplant..

[61] Wilhelmsson M., Glosli H., Ifversen M., Abrahamsson J., Winiarski J., Jahnukainen K., Hasle H., On behalf of the Nordic Society of Pediatric Hematology and Oncology (NOPHO) (2018). Long-term health outcomes in survivors of childhood AML treated with allogeneic HSCT: A NOPHO–AML Study. Bone Marrow Transplant..

[62] Uderzo C., Pillon M., Corti P., Tridello G., Tana F., Zintl F., Nysom K., Galambrun C., Fagioli F., Varotto S. (2007). Impact of cumulative anthracycline dose, preparative regimen and chronic graft-versus-host disease on pulmonary and cardiac function in children 5 years after allogeneic hematopoietic stem cell transplantation: A prospective evaluation on behalf of the EBMT Pediatric Diseases and Late Effects Working Parties. Bone Marrow Transplant..

[63] Armenian S.H., Sun C.-L., Shannon T., Mills G., Francisco L., Venkataraman K., Wong F.L., Forman S.J., Bhatia S. (2011). Incidence and predictors of congestive heart failure after autologous hematopoietic cell transplantation. Blood.

[64] Hochberg J.C., Cairo M.S., Friedman D.M. (2014). Cardio-Oncology Issues Among Pediatric Cancer and Stem Cell Transplant Survivors. Cardiol. Rev..

[65] Armenian S.H., Ding Y., Mills G., Sun C., Venkataraman K., Wong F.L., Neuhausen S.L., Senitzer D., Wang S., Forman S.J. (2013). Genetic susceptibility to anthracycline-related congestive heart failure in survivors of haematopoietic cell transplantation. Br. J. Haematol..

[66] Chow E.J., Baker K.S., Lee S.J., Flowers M.E., Cushing-Haugen K.L., Inamoto Y., Khera N., Leisenring W., Syrjala K.L., Martin P.J. (2014). Influence of Conventional Cardiovascular Risk Factors and Lifestyle Characteristics on Cardiovascular Disease After Hematopoietic Cell Transplantation. J. Clin. Oncol..

[67] Chaudhury S., Ayas M., Rosen C., Ma M., Viqaruddin M., Parikh S., Kharbanda S., Chiang K., Haight A., Bhatia M. (2017). A Multicenter Retrospective Analysis Stressing the Importance of Long-Term Follow-Up after Hematopoietic Cell Transplantation for β-Thalassemia. Biol. Blood Marrow Transplant..

[68] Armenian S.H., Bhatia S. (2008). Cardiovascular disease after hematopoietic cell transplantation-lessons learned. Haematologica.

[69] Tichelli A., Bhatia S., Socié G. (2008). Cardiac and cardiovascular consequences after haematopoietic stem cell transplantation. Br. J. Haematol..

[70] Tichelli A., Passweg J., Wojcik D., Harousseau J.-L., Masszi T., Zander A., Crawley C., Arat M., Sica S., Lutz P. (2008). Late cardiovascular events after allogeneic hematopoietic stem cell transplantation: A retrospective multicenter study of the Late Effects Working Party of the European Group for Blood and Marrow Transplantation. Haematologica.

[71] Tichelli A., Bucher C., Rovó A., Stussi G., Stern M., Paulussen M., Halter J., Meyer-Monard S., Heim D., Tsakiris D.A. (2007). Premature cardiovascular disease after allogeneic hematopoietic stem-cell transplantation. Blood.

[72] Mohty M., Malard F., Savani B.N. (2015). High-Dose Total Body Irradiation and Myeloablative Conditioning before Allogeneic Hematopoietic Cell Transplantation: Time to Rethink?. Biol. Blood Marrow Transplant..

[73] Kersting S., Verdonck L.F. (2008). Successful Outcome after Nonmyeloablative Allogeneic Hematopoietic Stem Cell Transplantation in Patients with Renal Dysfunction. Biol. Blood Marrow Transplant..

[74] Armenian S.H., Sun C.-L., Vase T., Ness K.K., Blum E., Francisco L., Venkataraman K., Samoa R., Wong F.L., Forman S.J. (2012). Cardiovascular risk factors in hematopoietic cell transplantation survivors: Role in development of subsequent cardiovascular disease. Blood.

[75] Vaksmann G., Nelken B., Deshildre A., Rey C. (2002). Pulmonary arterial occlusive disease following chemotherapy and bone marrow transplantation for leukaemia. Eur. J. Pediatr..

[76] Limsuwan A., Pakakasama S., Rochanawutanon M., Hong-Eng S. (2006). Pulmonary Arterial Hypertension after Childhood Cancer Therapy and Bone Marrow Transplantation. Cardiology.

[77] Shankar S., Choi J.K., Dermody T.S., Head D.R., Bunin N., Iannone R. (2004). Pulmonary Hypertension Complicating Bone Marrow Transplantation for Idiopathic Myelofibrosis. J. Pediatr. Hematol..

[78] Tanaka T.D., Ishizawar D.C., Saito T., Yoshii A., Yoshimura M. (2021). Successful treatment of pulmonary hypertension following hematopoietic stem cell transplant with a single oral tadalafil: A case report. Pulm. Circ..

[79] Versluys A.B., Bresters D., Information P.E.K.F.C. (2015). Pulmonary Complications of Childhood Cancer Treatment. Paediatr. Respir. Rev..

[80] Gower W.A., Collaco J.M., Mogayzel P.J. (2010). Lung Function and Late Pulmonary Complications Among Survivors of Hematopoietic Stem Cell Transplantation During Childhood. Paediatr. Respir. Rev..

[81] Tamburro R.F., Cooke K.R., Davies S.M., Goldfarb S., Hagood J.S., Srinivasan A., Steiner M.E., Stokes D., DiFronzo N., El-Kassar N. (2021). Pulmonary Complications of Pediatric Hematopoietic Cell Transplantation. A National Institutes of Health Workshop Summary. Ann. Am. Thorac. Soc..

[82] Yoshihara S., Yanik G., Cooke K.R., Mineishi S. (2007). Bronchiolitis Obliterans Syndrome (BOS), Bronchiolitis Obliterans Organizing Pneumonia (BOOP), and Other Late-Onset Noninfectious Pulmonary Complications following Allogeneic Hematopoietic Stem Cell Transplantation. Biol. Blood Marrow Transplant..

[83] Chow E.J., Cushing-Haugen K.L., Cheng G.-S., Boeckh M., Khera N., Lee S.J., Leisenring W., Martin P.J., Mueller B.A., Schwartz S.M. (2017). Morbidity and Mortality Differences Between Hematopoietic Cell Transplantation Survivors and Other Cancer Survivors. J. Clin. Oncol..

[84] Saglio F., Zecca M., Pagliara D., Giorgiani G., Balduzzi A., Calore E., Favre C., Faraci M., Prete A., Tambaro F.P. (2020). Occurrence of long-term effects after hematopoietic stem cell transplantation in children affected by acute leukemia receiving either busulfan or total body irradiation: Results of an AIEOP (Associazione Italiana Ematologia Oncologia Pediatrica) retrospective study. Bone Marrow Transplant..

[85] Inamoto Y., Lee S.J. (2017). Late effects of blood and marrow transplantation. Haematologica.

[86] Bergeron A., Cheng G.-S. (2017). Bronchiolitis Obliterans Syndrome and Other Late Pulmonary Complications After Allogeneic Hematopoietic Stem Cell Transplantation. Clin. Chest Med..

[87] Nieder M.L., McDonald G.B., Kida A., Hingorani S., Armenian S.H., Cooke K.R., Pulsipher M.A., Baker K.S. (2011). National Cancer Institute–National Heart, Lung and Blood Institute/Pediatric Blood and Marrow Transplant Consortium First International Consensus Conference on Late Effects After Pediatric Hematopoietic Cell Transplantation: Long-Term Organ Damage and Dysfunction. Biol. Blood Marrow Transplant..

[88] Au B.K., Au M.A., Chien J.W. (2011). Bronchiolitis Obliterans Syndrome Epidemiology after Allogeneic Hematopoietic Cell Transplantation. Biol. Blood Marrow Transplant..

[89] Socié G., Salooja N., Cohen A., Rovelli A., Carreras E., Locasciulli A., Korthof E., Weis J., Levy V., Tichelli A. (2003). Nonmalignant late effects after allogeneic stem cell transplantation. Blood.

[90] Rizzo J.D., Wingard J.R., Tichelli A., Lee S.J., Van Lint M.T., Burns L.J., Davies S.M., Ferrara J.L.M., Socié G. (2006). Recommended screening and preventive practices for long-term survivors after hematopoietic cell transplantation: Joint recommendations of the European Group for Blood and Marrow Transplantation, Center for International Blood and Marrow Transplant Research, and the American Society for Blood and Marrow Transplantation (EBMT/CIBMTR/ASBMT). Bone Marrow Transplant..

[91] Kim K., Lee H.J., Kim S., Lee J.W., Yoon J.-S., Chung N.G., Cho B. (2020). Lung Function Predicts Outcome in Children with Obstructive Lung Disease after Hematopoietic Stem Cell Transplantation. J. Pediatr. Hematol..

[92] Walther S., Rettinger E., Maurer H.M., Pommerening H., Jarisch A., Sörensen J., Schubert R., Berres M., Bader P., Zielen S. (2020). Long-term pulmonary function testing in pediatric bronchiolitis obliterans syndrome after hematopoietic stem cell transplantation. Pediatr. Pulmonol..

[93] Pham J., Rangaswamy J., Avery S., Borg B., Martin C., Munsif M., Lin T., Dabscheck E. (2020). Updated prevalence, predictors and treatment outcomes for bronchiolitis obliterans syndrome after allogeneic stem cell transplantation. Respir. Med..

[94] Freudenberger T.D., Madtes D.K., Curtis J.R., Cummings P., Storer B.E., Hackman R.C. (2003). Association between acute and chronic graft-versus-host disease and bronchiolitis obliterans organizing pneumonia in recipients of hematopoietic stem cell transplants. Blood.

[95] Palmas A., Tefferi A., Myers J.L., Scott J.P., Swensen S.J., Chen M.G., Gastineau D.A., Gertz M.A., Inwards D.J., Lacy M.Q. (1998). Late-onset noninfectious pulmonary complications after allogeneic bone marrow transplantation. Br. J. Haematol..

[96] Patriarca F., Skert C., Sperotto A., Damiani D., Cerno M., Geromin A., Zaja F., Stocchi R., Prosdocimo S., Fili’ C. (2004). Incidence, outcome, and risk factors of late-onset noninfectious pulmonary complications after unrelated donor stem cell transplantation. Bone Marrow Transplant..

[97] Coy D.L., Ormazabal A., Godwin J.D., Lalani T. (2005). Imaging Evaluation of Pulmonary and Abdominal Complications Following Hematopoietic Stem Cell Transplantation. RadioGraphics.

[98] Afessa B., Litzow M.R., Tefferi A. (2001). Bronchiolitis obliterans and other late onset non-infectious pulmonary complications in hematopoietic stem cell transplantation. Bone Marrow Transplant..

[99] Huang T.-T., Hudson M.M., Stokes D.C., Krasin M.J., Spunt S.L., Ness K.K. (2011). Pulmonary Outcomes in Survivors of Childhood Cancer. Chest.

[100] Limper A.H. (2004). Chemotherapy-induced lung disease. Clin. Chest Med..

[101] Meyer S., Reinhard H., Graf N. (2004). Pulmonary Dysfunction in Pediatric Oncology Patients. Pediatr. Hematol. Oncol..

[102] Abid S.H., Malhotra V., Perry M.C. (2001). Radiation-induced and chemotherapy-induced pulmonary injury. Curr. Opin. Oncol..

[103] Bruno B., Souillet G., Bertrand Y., Werck-Gallois M.C., Satta A.S., Bellon G. (2004). Effects of allogeneic bone marrow transplantation on pulmonary function in 80 children in a single paediatric centre. Bone Marrow Transplant..

[104] Mph P.A.H., Madtes D.K., Storer B.E., Sanders J.E. (2005). Pulmonary function in long-term survivors of pediatric hematopoietic cell transplantation. Pediatr. Blood Cancer.

[105] Inaba H., Yang J., Pan J., Stokes D., Krasin M.J., Srinivasan A., Hartford C.M., Pui C.-H., Leung W. (2010). Pulmonary dysfunction in survivors of childhood hematologic malignancies after allogeneic hematopoietic stem cell transplantation. Cancer.

[106] Cerveri I., Zoia M.C., Fulgoni P., Corsico A.G., Casali L., Tinelli C., Zecca M., Giorgiani G., Locatelli F. (1999). Late pulmonary sequelae after childhood bone marrow transplantation. Thorax.

[107] Leneveu H., Rubie H., Peyroulet M., Suc A., Robert A., Dutau G. (1999). Respiratory function in children undergoing bone marrow transplantation. Pediatr. Pulmonol..

[108] Socié G., Mary J.-Y., Esperou H., Robert D.V., Aractingi S., Ribaud P., Devergie A., Boudou P., Cathelinau B., Gluckman E. (2001). Health and functional status of adult recipients 1 year after allogeneic haematopoietic stem cell transplantation. Br. J. Haematol..

[109] Wieringa J., Van Kralingen K., Sont J., Bresters D. (2005). Pulmonary function impairment in children following hematopoietic stem cell transplantation. Pediatr. Blood Cancer.

[110] Stenehjem J.S., Smeland K.B., Murbraech K., Holte H., Kvaløy S.O., Wethal T., Kiserud C.E., Samersaw-Lund M.B. (2017). Obstructive and restrictive pulmonary dysfunction in long-term lymphoma survivors after high-dose therapy with autologous stem cell transplantation. Acta Oncol..

[111] Madanat-Harjuoja L.M., Valjento S., Vettenranta K., Kajosaari M., Dyba T., Taskinen M. (2014). Pulmonary function following allogeneic stem cell transplantation in childhood: A retrospective cohort study of 51 patients. Pediatr. Transplant..

[112] Fazekas T., Attarbaschi A., Lawitschka A., Seidel M., Pötschger U., Peters C., Mann G., Gadner H., Matthes-Martin S. (2012). Lethal Pulmonary Complications After Pediatric Allogeneic Hematopoietic Stem Cell Transplantation. Pediatr. Infect. Dis. J..

[113] Moermans C., Poulet C., Henket M., Bonnet C., Willems E., Baron F., Beguin Y., Louis R. (2013). Lung function and airway inflammation monitoring after hematopoietic stem cell transplantation. Respir. Med..

[114] Lee M.Y., Chiou T.J., Yang M.H., Bai L.Y., Hsiao L.T., Chao T.C., Tung S.-L., Wang W.S., Yen C.C., Liu J.H. (2005). Relatively favorable outcomes of post-transplant pulmonary function in patients with chronic myeloid leukemia receiving non-myeloablative allogeneic hematopoietic stem cell transplantation. Eur. J. Haematol..

[115] Chien J.W., Maris M.B., Sandmaier B.M., Maloney D.G., Storb R.F., Clark J.G. (2005). Comparison of lung function after myeloablative and 2 Gy of total body irradiation-based regimens for hematopoietic stem cell transplantation. Biol. Blood Marrow Transplant..

[116] Burt R.K., Oliveira M.C., Shah S.J., Moraes D.A., Simoes B., Gheorghiade M., Schroeder J., Ruderman E., Farge D., Chai Z.J. (2013). Cardiac involvement and treatment-related mortality after non-myeloablative haemopoietic stem-cell transplantation with unselected autologous peripheral blood for patients with systemic sclerosis: A retrospective analysis. Lancet.

[117] Oyama Y., Barr W.G., Statkute L., Corbridge T., Gonda E.A., Jovanovic B., Testori A., Burt R.K. (2007). Autologous non-myeloablative hematopoietic stem cell transplantation in patients with systemic sclerosis. Bone Marrow Transplant..

[118] Renaghan A.D., Jaimes E.A., Malyszko J., Perazella M.A., Sprangers B., Rosner M.H. (2019). Acute Kidney Injury and CKD Associated with Hematopoietic Stem Cell Transplantation. Clin. J. Am. Soc. Nephrol..

[119] Zager R., O’Quigley J., Zager B., Alpers C., Shulman H., Gamelin L., Stewart P., Thomas E. (1989). Acute Renal Failure Following Bone Marrow Transplantation: A Retrospective Study of 272 Patients. Am. J. Kidney Dis..

[120] Parikh C.R., Schrier R.W., Storer B., Diaconescu R., Sorror M.L., Maris M.B., Maloney D.G., McSweeney P., Storb R., Sandmaier B.M. (2005). Comparison of ARF after myeloablative and nonmyeloablative hematopoietic cell transplantation. Am. J. Kidney Dis..

[121] Verghese P.S., Finn L.S., Englund J.A., Sanders J.E., Hingorani S.R. (2009). BK nephropathy in pediatric hematopoietic stem cell transplant recipients. Pediatr. Transplant..

[122] Ho V.T., Cutler C., Carter S., Martin P., Adams R., Horowitz M., Ferrara J., Soiffer R., Giralt S. (2005). Blood and Marrow Transplant Clinical Trials Network Toxicity Committee Consensus Summary: Thrombotic Microangiopathy after Hematopoietic Stem Cell Transplantation. Biol. Blood Marrow Transplant..

[123] Hingorani S., Guthrie K.A., Schoch G., Weiss N.S., McDonald G.B. (2007). Chronic kidney disease in long-term survivors of hematopoietic cell transplant. Bone Marrow Transplant..

[124] Clavert A., Peric Z., Brissot E., Malard F., Guillaume T., Delaunay J., Dubruille V., Le Gouill S., Mahe B., Gastinne T. (2016). Late Complications and Quality of Life after Reduced-Intensity Conditioning Allogeneic Stem Cell Transplantation. Biol. Blood Marrow Transplant..

[125] Gifford G., Milliken S., Greenfield J. (2013). Diabetic ketoacidosis secondary to L-asparaginase in acute lymphoblastic leukaemia. Intern. Med. J..

[126] Al-Hazzouri A., Cao Q., Burns L.J., Weisdorf D.J., Majhail N.S. (2008). Similar Risks for Chronic Kidney Disease in Long-Term Survivors of Myeloablative and Reduced-Intensity Allogeneic Hematopoietic Cell Transplantation. Biol. Blood Marrow Transplant..

[127] Weiss A.S., Sandmaier B.M., Storer B., Storb R., McSweeney P.A., Parikh C.R. (2005). Chronic Kidney Disease Following Non-Myeloablative Hematopoietic Cell Transplantation. Am. J. Transplant..

[128] Choi M., Sun C.-L., Kurian S., Carter A., Bs L.F., Forman S.J., Bhatia S. (2008). Incidence and predictors of delayed chronic kidney disease in long-term survivors of hematopoietic cell transplantation. Cancer.

[129] Hingorani S. (2016). Renal Complications of Hematopoietic-Cell Transplantation. N. Engl. J. Med..

[130] Delgado J., Cooper N., Thomson K., Duarte R., Jarmulowicz M., Cassoni A., Kottaridis P., Peggs K., Mackinnon S. (2006). The Importance of Age, Fludarabine, and Total Body Irradiation in the Incidence and Severity of Chronic Renal Failure after Allogeneic Hematopoietic Cell Transplantation. Biol. Blood Marrow Transplant..

[131] Frisk P., Bratteby L., Carlson K., Lönnerholm G. (2002). Renal function after autologous bone marrow transplantation in children: A long-term prospective study. Bone Marrow Transplant..

[132] Deconinck E., Kribs M., Rebibou J.-M., Bulabois C.E., Ducloux D., Cahn J.-Y. (2005). Cytomegalovirus infection and chronic graft-versus-host disease are significant predictors of renal failure after allogeneic hematopoietic stem cell transplantation. Haematologica.

[133] Holthe J.E.K.-V., Bresters D., Ahmed-Ousenkova Y.M., Goedvolk C.A., Abbink F.C.H., Wolterbeek R., Bredius R.G.M., Pauwels E.K.J., van der Heijden A.J. (2005). Long-term renal function after hemopoietic stem cell transplantation in children. Bone Marrow Transplant..

[134] Kersting S., Hené R.J., Koomans H.A., Verdonck L.F. (2007). Chronic Kidney Disease after Myeloablative Allogeneic Hematopoietic Stem Cell Transplantation. Biol. Blood Marrow Transplant..

[135] Wu N.L., Hingorani S., Cushing-Haugen K.L., Lee S.J., Chow E.J. (2021). Late Kidney Morbidity and Mortality in Hematopoietic Cell Transplant Survivors. Transplant. Cell. Ther..

[136] Lugthart G., Jordans C.C., de Pagter A.P., Bresters D., der Zijde C.M.J.-V., Bense J.E., van Rooij-Kouwenhoven R.W., Sukhai R.N., Louwerens M., Dorresteijn E.M. (2021). Chronic kidney disease ten years after pediatric allogeneic hematopoietic stem cell transplantation. Kidney Int..

[137] Sakaguchi M., Nakayama K., Yamaguchi H., Mii A., Shimizu A., Inai K., Onai D., Marumo A., Omori I., Yamanaka S. (2019). Risk Factors for Acute Kidney Injury and Chronic Kidney Disease following Allogeneic Hematopoietic Stem Cell Transplantation for Hematopoietic Malignancies. Acta Haematol..

[138] Gutiérrez-García G., Villarreal J., Garrote M., Rovira M., Blasco M., Suárez-Lledó M., Rodríguez-Lobato L.G., Charry P., Rosiñol L., Marín P. (2020). Impact of severe acute kidney injury and chronic kidney disease on allogeneic hematopoietic cell transplant recipients: A retrospective single center analysis. Bone Marrow Transplant..

[139] Farhadfar N., Dias A., Wang T., Fretham C., Chhabra S., Murthy H.S., Broglie L., D’Souza A., Gadalla S.M., Gale R.P. (2021). Impact of Pretransplantation Renal Dysfunction on Outcomes after Allogeneic Hematopoietic Cell Transplantation. Transplant. Cell. Ther..

[140] Srinivasan R., Balow J.E., Sabnis S., Lundqvist A., Igarashi T., Takahashi Y., Austin H., Tisdale J., Barrett J., Geller N. (2005). Nephrotic syndrome: An under-recognised immune-mediated complication of non-myeloablative allogeneic haematopoietic cell transplantation. Br. J. Haematol..

[141] Colombo A.A., Rusconi C., Esposito C., Bernasconi P., Caldera D., Lazzarino M., Alessandrino E.P. (2006). Nephrotic Syndrome after Allogeneic Hematopoietic Stem Cell Transplantation as a Late Complication of Chronic Graft-versus-Host Disease. Transplantation.

[142] Pegelow C.H., Colangelo L., Steinberg M., Wright E.C., Smith J., Phillips G., Vichinsky E. (1997). Natural History of Blood Pressure in Sickle Cell Disease: Risks for Stroke and Death Associated with Relative Hypertension in Sickle Cell Anemia. Am. J. Med..

[143] Klings E.S., Machado R., Barst R.J., Morris C.R., Mubarak K.K., Gordeuk V.R., Kato G., Ataga K.I., Gibbs J.S., Castro O. (2014). An Official American Thoracic Society Clinical Practice Guideline: Diagnosis, Risk Stratification, and Management of Pulmonary Hypertension of Sickle Cell Disease. Am. J. Respir. Crit. Care Med..

[144] Parent F., Bachir D., Inamo J., Lionnet F., Driss F., Loko G., Habibi A., Bennani S., Savale L., Adnot S. (2011). A Hemodynamic Study of Pulmonary Hypertension in Sickle Cell Disease. N. Engl. J. Med..

[145] Mehari A., Alam S., Tian X., Cuttica M.J., Barnett C.F., Miles G., Xu D., Seamon C., Adams-Graves P., Castro O.L. (2013). Hemodynamic Predictors of Mortality in Adults with Sickle Cell Disease. Am. J. Respir. Crit. Care Med..

[146] Sachdev V., Machado R., Shizukuda Y., Rao Y.N., Sidenko S., Ernst I., Peter M.S., Coles W.A., Rosing D.R., Blackwelder W.C. (2007). Diastolic Dysfunction Is an Independent Risk Factor for Death in Patients with Sickle Cell Disease. J. Am. Coll. Cardiol..

[147] Maitra P., Caughey M., Robinson L., Desai P.C., Jones S., Nouraie M., Gladwin M.T., Hinderliter A., Cai J., Ataga K.I. (2017). Risk factors for mortality in adult patients with sickle cell disease: A meta-analysis of studies in North America and Europe. Haematologica.

[148] Stenger E., Khemani K., Ross D., Meacham L., McCracken C., Haight A., Krishnamurti L. (2017). Increased Proportion of Sickle Cell Disease Patients Develop Cardiovascular Disease Risk Factors Following Successful Hematopoietic Cell Transplantation. Blood.

[149] Pedersen S.J.V., Monagel D.A., Mammen C., Lewis V.A., Guilcher G.M.T., Bruce A.A. (2020). Stable renal function in children and adolescents with sickle cell disease after nonmyeloablative hematopoietic stem cell transplantation. Pediatr. Blood Cancer.

[150] Stenger E., Abraham A., Travers C.D., Rangarajan H.G., Hsu D., Chaudhury S., Kasow K.A., Eckrich M.J., Ngwube A.I., Kanter J. (2017). Development of a Multi-Center Cohort to Evaluate Long-Term Outcomes and Late Effects Following Hematopoietic Cell Transplantation for Sickle Cell Disease: A STAR Initiative. Blood.

[151] Stenger E., Phelan M.R., Shaw B.E., Battiwalla M., Bo-Subait M.S., Brazauskas R., Buchbinder M.D.K., Hamilton B.K., Shenoy S., Krishnamurti L. (2019). Excellent Overall Survival and Low Incidence of Late Effects in Patients Undergoing Allogeneic Hematopoietic Cell Transplant for Sickle Cell Disease: A Report from the Center for International Blood and Marrow Transplant Research (CIBMTR). Blood.

[152] Dallas M.H., Triplett B., Shook D.R., Hartford C., Srinivasan A., Laver J., Ware R., Leung W. (2013). Long-Term Outcome and Evaluation of Organ Function in Pediatric Patients Undergoing Haploidentical and Matched Related Hematopoietic Cell Transplantation for Sickle Cell Disease. Biol. Blood Marrow Transplant..

[153] Friedman D., Dozor A.J., Milner J., D’Souza M., Talano J.-A., Moore T.B., Shenoy S., Shi Q., Walters M.C., Vichinsky E. (2021). Stable to improved cardiac and pulmonary function in children with high-risk sickle cell disease following haploidentical stem cell transplantation. Bone Marrow Transplant..

[154] Sachdev V., Hsieh M., Jeffries N., Noreuil A., Li W., Sidenko S., Hannoush H., Limerick E., Wilson D., Tisdale J. (2019). Reversal of a rheologic cardiomyopathy following hematopoietic stem cell transplantation for sickle cell disease. Blood Adv..

[155] Covi S., Ravindranath Y., Farooqi A., Savasan S., Chu R., Aggarwal S. (2017). Changes in Bi-ventricular Function after Hematopoietic Stem Cell Transplant as Assessed by Speckle Tracking Echocardiography. Pediatr. Cardiol..

[156] Bhatia M., Jin Z., Baker C.V.H., Geyer M., Radhakrishnan K., Morris E., Satwani P., George D.M.M.S., Garvin J.H., Del Toro G. (2014). Reduced toxicity, myeloablative conditioning with BU, fludarabine, alemtuzumab and SCT from sibling donors in children with sickle cell disease. Bone Marrow Transplant..

[157] Gladwin M.T., Sachdev V., Jison M.L., Shizukuda Y., Plehn J.F., Minter K., Brown B., Coles W.A., Nichols J.S., Ernst I. (2004). Pulmonary Hypertension as a Risk Factor for Death in Patients with Sickle Cell Disease. N. Engl. J. Med..

[158] Vendrame F., Olops L., Saad S.T.O., Costa F.F., Fertrin K.Y. (2019). Hypocholesterolemia and dysregulated production of angiopoietin-like proteins in sickle cell anemia patients. Cytokine.

[159] Kassim A.A., Payne A.B., Rodeghier M., Macklin E.A., Strunk R.C., DeBaun M.R. (2015). Low forced expiratory volume is associated with earlier death in sickle cell anemia. Blood.

[160] Walters M.C., Hardy K., Edwards S., Adamkiewicz T., Barkovich J., Bernaudin F., Buchanan G.R., Bunin N., Dickerhoff R., Giller R. (2010). Pulmonary, Gonadal, and Central Nervous System Status after Bone Marrow Transplantation for Sickle Cell Disease. Biol. Blood Marrow Transplant..

[161] Mynarek M., Riehm C.B.D.C., Brinkmann F., Weißenborn K., Tell-Lüersen M., Heuft H.-G., Maecker-Kolhoff B., Sykora K.-W. (2013). Normalized Transcranial Doppler Velocities, Stroke Prevention and Improved Pulmonary Function after Stem Cell Transplantation in Children with Sickle Cell Anemia. Klin Padiatr..

[162] Krishnamurti L., Kharbanda S., Biernacki M.A., Zhang W., Baker K.S., Wagner J.E., Wu C.J. (2008). Stable Long-Term Donor Engraftment following Reduced-Intensity Hematopoietic Cell Transplantation for Sickle Cell Disease. Biol. Blood Marrow Transplant..

[163] Matthes-Martin S., Lawitschka A., Fritsch G., Lion T., Grimm B., Breuer S., Boztug H., Karlhuber S., Holter W., Peters C. (2013). Stem cell transplantation after reduced-intensity conditioning for sickle cell disease. Eur. J. Haematol..

[164] Vichinsky E., Hoppe C.C., Ataga K.I., Ware R.E., Nduba V., El-Beshlawy A., Hassab H., Achebe M.M., Al Kindi S., Brown R.C. (2019). A Phase 3 Randomized Trial of Voxelotor in Sickle Cell Disease. N. Engl. J. Med..

[165] Niihara Y., Miller S.T., Kanter J., Lanzkron S., Smith W.R., Hsu L.L., Gordeuk V.R., Viswanathan K., Sarnaik S., Osunkwo I. (2018). A Phase 3 Trial ofl-Glutamine in Sickle Cell Disease. N. Engl. J. Med..

[166] Ataga K.I., Kutlar A., Kanter J., Liles D., Cancado R., Friedrisch J., Guthrie T.H., Knight-Madden J., Alvarez O.A., Gordeuk V.R. (2017). Crizanlizumab for the Prevention of Pain Crises in Sickle Cell Disease. N. Engl. J. Med..

